# Supplementary material for: School-Based Nutrition Interventions in Children Aged 6 to 18 Years: An Umbrella Review of Systematic Reviews
Source: Nutrients. 2021 Nov 17;13(11):4113. doi: 10.3390/nu13114113 (PMC8618558; doi:10.3390/nu13114113)
Supplement: Supplementary file 1 [file nutrients-13-04113-s001.zip › Supplementary files_clean.pdf]

Supplementary files

Table S1. Search strategy Database(s): Medline 1946 – present.

#	Searches	Results
1	child/ or Adolescent/	2836436
2	((high or middle or junior or primary or elementary or senior or prep*) adj2 (school* or student*)).mp.	70705
3	(child* or school?age* or adolescen* or teen*).tw.	1574723
4	1 or 2 or 3	3448445
5	health behavior/ or body weight/ or overweight/ or obesity/ or body composition/ or waist circumference/ or anthropometry/ or beverages/ or carbonated beverages/ or fruit/ or vegetables/ or skinfold thickness/	533701
6	(health?behavio* or Cogn* or behavio* or learning or attitud* or stigma).tw.	1919058
7	(Eating?behavio* or eat* or diet* or fruit* or vegetable* or sugar?sweetened or drink* or beverage* or soft?drink* or water or food* or nutrition or energy?dens* or sugar or energy?rich or portion?size or fast?food* or nutrient?assessment).tw.	2029051
8	(body?weight or weight or overweight or obes* or BMI or Body?Mass?Index or waist?circumference or anthropomet* or body?composition or skinfold* or high?weight or fat*).tw.	1870850
9	5 or 6 or 7 or 8	5213397
10	mass media/ or diet/ or television/ or meals/ or food/ or cell phone/	225511
11	(cafeteria* or canteen* or cafe* or school?lunch* or school?meal? or food or feeding?program or food?service* or diet* or meal? or nutrition or purchas* or sold or bought or buy or school*).tw.	1353397
12	(mass?media or campaign* or consumer?research or (health adj (communication or information)) or advertis* or marketing or social?media).tw.	107727
13	(screen?viewing or television or tv or video?games or computer or screen?media or mobile?phone or cell?phone or electronic or technolo*).tw.	934025
14	10 or 11 or 12 or 13	2380153
15	(((comprehensive* or integrative or systematic*) adj3 (bibliographic* or review* or literature)) or (meta-analy* or metaanaly* or "research synthesis" or ((information or data) adj3 synthesis) or (data adj2 extract*))).ti,ab. or (cinahl or (cochrane adj3 trial*) or embase or medline or psyclit or (psycinfo not "psycinfo database") or pubmed or scopus or "sociological abstracts").ab. or ("cochrane database" or evidence report technology assessment or evidence report technology assessment summary).jn. or Evidence Report: Technology Assessment*.jn. or ((review adj5 (rationale or evidence)).ti,ab. and review.pt.) or meta-analysis as topic/ or Meta-Analysis.pt.	489135
16	("clinical trial" or "clinical trial, phase i" or "clinical trial, phase ii" or clinical trial, phase iii or clinical trial, phase iv or controlled clinical trial or "multicenter study" or "randomized controlled trial").pt. or double-blind method/ or clinical trials as topic/ or controlled clinical?trials as topic/ or randomized?controlled?trials as topic/ or early termination of clinical trials as topic/ or multicenter studies as topic/ or ((randomi?ed adj7 trial*) or (controlled adj3 trial*) or (clinical adj2 trial*) or ((single or doubl* or tripl* or treb*) and (blind* or mask*))).ti,ab,kw. or ("4 arm" or "four?arm").ti,ab,kw.	1618844

17	cohort studies/ or longitudinal studies/ or follow-up studies/ or prospective studies/ or retrospective studies/ or cohort.mp. or longitudinal.mp. or prospective.mp. or retrospective.mp.	2726476
18	16 or 17	3892382
19	4 and 9 and 14 and 15 and 18	3753
**20**	**limit 19 to (english language and yr="2010 -Current")**	**2929**

Table S2. Overlap matrix of the RCTs of school-based nutrition interventions reporting a dietary intake outcome.

	Education		Environment			HPS*	Other						
**Primary study ID**	**Meiklejohn 2016** [1]	**Rahman 2017** [2]	**Bonell 2013** [3]	**Micha 2018** [4]	**Pineda 2021** [5]	**Langford 2014** [6]	**Champion 2019** [7]	**Delgado-Noguera 2011** [8]	**Evans 2012** [9]**	**MacArthur 2018** [10]	**Nally 2021** [11]**	**Rose 2021** [12]	**Singhal 2020** [13]
Aceves-Martins 2017												FV	
Akdemir 2017													Diet in general
Alvirde-García 2013													Diet in general
Amaro 2006								FV					
Anderson 2005				Fruit, vege, FV	Fruit, vege	Fruit, fat							
Ashfield-Watt 2009				Fruit	Fruit								
Baranowski 2000								FV					
Baranowski 2003								FV					
Bayer 2009		SSB											
Bere 2005				FV									
Bere 2006a				FV				FV					
Bere 2006b	FV					Fruit							
Bere 2010					Fruit								
Bere 2015					Fruit								
Bessems 2012												FV	
Birnbaum 2002	FV, fat												
Bjelland 2011	SSB	SSB											
Bush 1989										Diet in general			
Caballero 2003						Fat							
Carfora 2016												FV	

	**Education**		**Environment**			**HPS***	**Other**						
**Primary study ID**	**Meiklejohn 2016** [1]	**Rahman 2017** [2]	**Bonell 2013** [3]	**Micha 2018** [4]	**Pineda 2021** [5]	**Langford 2014** [6]	**Champion 2019** [7]	**Delgado-Noguera 2011** [8]	**Evans 2012** [9]**	**MacArthur 2018** [10]	**Nally 2021** [11]**	**Rose 2021** [12]	**Singhal 2020** [13]
Casazza 2007							FV, fat						
Cohen 2015					Vege								
Coleman 2012				SSB									
Colín-Ramírez 2010						Fat							
Crepinsek 2006					Vege								
Crespo 2012						Fruit							
Cunha 2013		SSB											Diet in general
Day 2008					Fruit								
Dzewaltowski 2009			Diet in general										
Eriksen 2003					Vege								
Evans 2013						Fruit, fat							
Ezendam 2012		SSB					FV						
Foster 2008	Unclear				Fruit	Fruit, fat							
Foster 2010						Fruit, fat							
Gortmaker 1999					Fruit								
Habib-Mourad 2013													Diet in general
Haerens 2006	Fruit, fat, SSB					Fruit, fat	FV, fat						
Haerens 2007	Fruit, fat, SSB	SSB					Fat						
He 2009				FV									
Hoppu 2010	Fruit, FV			Fruit		Fruit, fat						FV, SSB	
James 2004		SSB											
Keshani 2016													Diet in general

	Education		Environment			HPS*	Other						
**Primary study ID**	**Meiklejohn 2016** [1]	**Rahman 2017** [2]	**Bonell 2013** [3]	**Micha 2018** [4]	**Pineda 2021** [5]	**Langford 2014** [6]	**Champion 2019** [7]	**Delgado-Noguera 2011** [8]	**Evans 2012** [9]**	**MacArthur 2018** [10]	**Nally 2021** [11]**	**Rose 2021** [12]	**Singhal 2020** [13]
Kobel 2014		SSB											
Levy 2012						Fat							
Li 2017													Diet in general
Liu 2018													Diet in general
Luepker 1998						Fat							
Lytle 2004a	FV, fat			Fruit, vege, FV		Fruit, fat							
Lytle 2004b					Fruit, vege								
Mangunkusumo 2007								FV					
Mauriello 2010							FV						
Mihas 2010	Fruit, diet, fat											Sat fat, energy, fruit	
Millar 2011	Diet in general												
Moore 2008				Fruit, unhealthy snacks	Fruit			FV					
Muckelbauer 2009		SSB											
Nader 1999										Diet in general			
Nicklas 1996				Fat, sat fat, calories									
Pannunzio 2007								FV					
Perry 1998						Fruit, fat		FV					
Perry 2004								FV					

	Education		Environment			HPS*	Other						
**Primary study ID**	**Meiklejohn 2016** [1]	**Rahman 2017** [2]	**Bonell 2013** [3]	**Micha 2018** [4]	**Pineda 2021** [5]	**Langford 2014** [6]	**Champion 2019** [7]	**Delgado-Noguera 2011** [8]	**Evans 2012** [9]**	**MacArthur 2018** [10]	**Nally 2021** [11]**	**Rose 2021** [12]	**Singhal 2020** [13]
Prell 2005	Fish												
Prochaska 2004							FV						
Ransley 2007					Vege								
Rees 2010												FV	
Reinaerts 2007								FV					
Reynolds 2000						Fruit, fat		FV					
Rosario 2013		SSB											
Sahota 2001						Fruit, fat		FV					
Sallis 2003			Diet in general			Fat							
Saraf 2015										Diet in general			
Sgambato 2016													Diet in general
Sichieri 2009		SSB											Diet in general
Siega-Riz 2011		SSB											
Singh 2009		SSB										SSB	
Te Velde 2008	Fruit, FV			Fruit, vege, FV	Fruit, vege	Fruit		FV					
Trevino 2004						Fat							
van Nassau 2014												SSB, breakfast	
Velicer 2013							FV						
Viggiano 2015												FV	
Williamson 2007				Fat, sat fat, calories									
Williamson 2012						Fat							

	Education		Environment			HPS*	Other						
**Primary study ID**	**Meiklejohn 2016** [1]	**Rahman 2017** [2]	**Bonell 2013** [3]	**Micha 2018** [4]	**Pineda 2021** [5]	**Langford 2014** [6]	**Champion 2019** [7]	**Delgado-Noguera 2011** [8]	**Evans 2012** [9]**	**MacArthur 2018** [10]	**Nally 2021** [11]**	**Rose 2021** [12]	**Singhal 2020** [13]
Xu 2014													Diet in general

Abbreviations: FV fruit and/or vegetable; HPS Health Promoting School; Sat fat saturated fat; SSB sugar-sweetened beverage; Vege vegetable. *School interventions based on the Health Promoting Schools (HPS) framework, with active components in school education, environment and partnerships. **Evans 2012 and Nally 2021 did not provide enough information to be able to identify which of the primary studies were included in the meta-analyses.

Table S3. Methodological quality assessment for each systematic review, according to the AMSTAR2 scale.

Reference	1	2	3	4	5	6	7	8	9a	9b	10	11a	11b	12	13	14	15	16
Bonell 2013 [3]	Y	Y	N	PY	Y	Y	N	Y	N	NA	N	NMA	NA	NMA	Y	Y	NMA	Y
Champion 2019 [7]	Y	Y	N	PY	Y	Y	N	Y	Y	NA	N	Y	NA	N	Y	Y	N	Y
Delgado-Noguera 2011 [8]	Y	N	N	PY	N	N	N	PY	N	NA	N	Y	NA	Y	Y	Y	N	Y
Evans 2012 [9]	Y	N	N	PY	Y	N	N	N	PY	NA	N	Y	NA	N	N	Y	Y	Y
Langford 2014 [6]	Y	Y	Y	PY	Y	Y	Y	Y	Y	NA	Y	Y	NA	Y	Y	Y	Y	Y
MacArthur 2018 [10]	Y	Y	Y	Y	Y	Y	Y	Y	Y	NA	Y	Y	NA	Y	Y	Y	Y	Y
Meiklejohn 2016 [1]	Y	N	N	N	N	Y	N	N	N	NA	N	NMA	NA	NMA	N	N	NMA	Y
Micha 2018 [4]	Y	PY	N	PY	Y	Y	N	N	N	NA	N	Y	NA	Y	N	Y	N	Y
Nally 2021 [11]	Y	Y	N	N	Y	N	N	Y	Y	NA	N	Y	NA	Y	N	Y	Y	Y
Pineda 2021 [5]	Y	Y	N	N	Y	N	N	PY	Y	NA	N	N	NA	N	Y	Y	Y	Y
Rahman 2017 [2]	Y	N	Y	N	Y	Y	Y	Y	Y	NA	Y	Y	NA	N	N	Y	Y	Y
Rose 2021 [12]	Y	PY	N	PY	N	Y	Y	PY	PY	NA	N	NMA	NA	NMA	N	N	NMA	Y
Singhal 2020 [13]	Y	PY	N	PY	N	N	N	Y	Y	NA	N	NMA	NA	NMA	Y	N	NMA	Y

Abbreviation: Y yes; N no; PY partial yes; NMA no meta-analysis conducted. (1) Did the research questions and inclusion criteria for the review include the components of PICO (Population, Intervention, Comparator, Outcome)? (2) Did the report of the review contain an explicit statement that the review methods were established prior to the conduct of the review and did the report justify any significant deviations from the protocol? (3) Did the review authors explain their selection of the study designs for inclusion in the review? (4) Did the review authors use a comprehensive literature search strategy? (5) Did the review authors perform study selection in duplicate? (6) Did the review authors perform data extraction in duplicate? (7) Did the review authors provide a list of excluded studies and justify the exclusions? (8) Did the review authors describe the included studies in adequate detail? (9) Did the review authors use a satisfactory technique for assessing the risk of bias (RoB) in individual studies that were included in the review? (9a) Randomised controlled trials (RCTs) (9b) non-randomized studies of interventions (NRSI) (10) Did the review report on the sources of funding for the studies included in the review? (11) If meta-analysis was performed did the review authors use appropriate methods for statistical combination of results? (11a) RCTs (11b) NRSI (12) If meta-analysis was performed, did the review authors assess the potential impact of RoB in individual studies on the results of the meta-analysis or other evidence synthesis? (13) Did the review authors account for RoB in individual studies when interpreting/discussing the results of the review? (14) Did the review authors provide a satisfactory explanation for, and discussion of, any heterogeneity observed in the results of the review? (15) If they performed quantitative synthesis did the review authors carry out an adequate investigation of publication bias (small study bias) and discuss its likely impact on the results of the review? (16) Did the review authors report any potential sources of conflict of interest, including any funding they received for conducting the review? The overall confidence in the results of the systematic reviews proposed by the AMSTAR2 tool is? defined as: (1) high—No, or one non-critical weakness: the systematic review provides an accurate and comprehensive summary of the results; (2) moderate—more than one non-critical weakness but no critical flaws: the systematic review provides an accurate summary of the results; (3) low—one critical flaw, with or without non-critical weaknesses: the systematic review may not provide an accurate and comprehensive summary of the results; (4) critically low—more than one critical flaw, with or with-out non-critical weaknesses: the review should not be relied on to provide an accurate and comprehensive summary of the results.

References

1. Meiklejohn, S.; Ryan, L.; Palermo, C. A Systematic Review of the Impact of Multi-Strategy Nutrition Education Programs on Health and Nutrition of Adolescents. *J. Nutr. Educ. Behav.* **2016**, *48*, 631-646. e1, doi:10.1016/j.jneb.2016.07.015.
2. Rahman, AA.; Lamis, J.; Kahale, LA.; Adair, P.; Pine, C. Effectiveness of Behavioral Interventions to Reduce the Intake of Sugar-Sweetened Beverages in Children and Adolescents: A Systematic Review and Meta-Analysis. *Nutr. Rev.* **2017**, *76*, 88–107, doi:10.1093/nutrit/nux061.
3. Bonell, C.; Jamal, F.; Harden, A.; Wells, H.; Parry, W.; Fletcher, A.; Petticrew, M.; Thomas, J.; Whitehead, M.; Campbell, R.; et al. Systematic Review of the Effects of Schools and School Environment Interventions on Health: Evidence Mapping and Synthesis. **2013**, *1*, doi:10.3310/phr01010.
4. Micha, R.; Karageorgou, D.; Bakogianni, I.; Trichia, E.; Whitsel, LP.; Story, M.; Peñalvo, JL.; Mozaffarian, D. Effectiveness of School Food Environment Policies on Children's Dietary Behaviors: A Systematic Review and Meta-Analysis. *PLoS One* **2018**, *13*, e0194555, doi:10.1371/journal.pone.0194555.
5. Pineda, E.; Bascunan, J.; Sassi, F. Improving the School Food Environment for the Prevention of Childhood Obesity: What Works and What Doesn't. *Obes. Rev.* **2021**, *22*, doi:10.1111/obr.13176.
6. Langford, R.; Bonell, C.P.; Jones, H.E.; Pouliou, T.; Murphy, S.M.; Waters, E.; Komro, K.A.; Gibbs, L.F.; Magnus, D.; Campbell, R. The WHO Health Promoting School Framework for Improving the Health and Well-Being of Students and Their Academic Achievement. *Cochrane Database Syst. Rev.* **2014**, CD008958, doi:10.1002/14651858.CD008958.pub2.
7. Champion, KE.; Parmenter, B.; McGowan, C.; Spring, B.; Wafford, QE.; Gardner, LA.; Thornton, L.; McBride, N.; Barrett, EL.; Teesson, M. Effectiveness of School-Based EHealth Interventions to Prevent Multiple Lifestyle Risk Behaviours among Adolescents: A Systematic Review and Meta-Analysis. *Lancet Digit. Health* **2019**, *1*, e206–e221, doi:10.1016/S2589-7500(19)30088-3.
8. Delgado-Noguera, M.; Tort, S.; Martinez-Zapata, MJ.; Bonfill, X. Primary School Interventions to Promote Fruit and Vegetable Consumption: A Systematic Review and Meta-Analysis. *Prev. Med.* **2011**, *53*, 3–9, doi:10.1016/j.ypmed.2011.04.016.
9. Evans, CE.; Christian, MS.; Cleghorn, CL.; Greenwood, DC.; Cade, JE. Systematic Review and Meta-Analysis of School-Based Interventions to Improve Daily Fruit and Vegetable Intake in Children Aged 5 to 12 y. *Am J Clin Nutr* **2012**, *96*, 889–901, doi:10.3945/ajcn.111.030270.
10. MacArthur, G.; Caldwell, DM.; Redmore, J.; Watkins, SH.; Kipping, R.; White, J.; Chittleborough, C.; Langford, R.; Er, V.; Lingam, R.; et al. Individual-, Family-, and School-Level Interventions Targeting Multiple Risk Behaviours in Young People. *Cochrane Database Syst. Rev.* **2018**, *10*, CD009927, doi:10.1002/14651858.CD009927.pub2.
11. Nally, S.; Carlin, A.; Blackburn, N.E.; Baird, J.S.; Salmon, J.; Murphy, M.H.; Gallagher, A.M. The Effectiveness of School-Based Interventions on Obesity-Related Behaviours in Primary School Children: A Systematic Review and Meta-Analysis of Randomised Controlled Trials. *Child. Basel Switz.* **2021**, *8*, 489, doi:10.3390/children8060489.
12. Rose, K.; O'Malley, C.; Eskandari, F.; Lake, A.A.; Brown, L.; Ells, L.J. The Impact of, and Views on, School Food Intervention and Policy in Young People Aged 11–18 Years in Europe: A Mixed Methods Systematic Review. *Obes. Rev.* **2021**, *22*, e13186-n/a, doi:10.1111/obr.13186.
13. Singhal, J.; Herd, C.; Adab, P.; Pallan, M. Effectiveness of School-Based Interventions to Prevent Obesity among Children Aged 4 to 12 Years Old in Middle-Income Countries: A Systematic Review and Meta-Analysis. *Obes. Rev.* **2020**, *28*, doi:10.1111/obr.13105.
